# Effects of Ultrasound-Assisted Extraction and Solvent on the Phenolic Profile, Bacterial Growth, and Anti-Inflammatory/Antioxidant Activities of Mediterranean Olive and Fig Leaves Extracts

**DOI:** 10.3390/molecules25071718

**Published:** 2020-04-09

**Authors:** Cristina Alcántara, Tihana Žugčić, Radhia Abdelkebir, Jose V. García-Pérez, Anet Režek Jambrak, José M. Lorenzo, María Carmen Collado, Daniel Granato, Francisco J. Barba

**Affiliations:** 1 Department of Biotechnology, Institute of Agrochemistry and Food Technology, Spanish National Research Council (IATA-CSIC), Av. Agustin Escardino 7, 46980 Valencia, Spain; crisalba@iata.csic.es; 2 Faculty of Food Technology and Biotechnology, University of Zagreb, Pierottijeva 6, 10000 Zagreb, Croatia; tihana.zugcic@gmail.com (T.Ž.); arezek@pbf.hr (A.R.J.); 3 Nutrition and Food Science Area, Preventive Medicine and Public Health, Food Science, Toxicology and Forensic Medicine Department, Universitat de València, Avda. Vicent Andrés Estellés, 46100 València, Spain; abdelkebirradhia@yahoo.fr; 4 Range Ecology Laboratory in the Institute of Arid Regions (IRA) of Medenine, 4100 Medenine, Tunisia; 5 Grupo de Análisis y Simulación de Procesos Agroalimentarios (ASPA), Departamento de Tecnología de Alimentos, Universitat Politècnica de València, 46022 Valencia, Spain; jogarpe4@tal.upv.es; 6 Centro Tecnológico de la Carne de Galicia, Adva. Galicia n° 4, Parque Tecnológico de Galicia, San Cibrao das Viñas, 32900 Ourense, Spain; jmlorenzo@ceteca.net; 7 Food Processing and Quality, Production Systems Unit-Natural Resources Institute Finland (Luke)-Tietotie 2, FI-02150 Espoo, Finland

**Keywords:** bioactive compounds, extraction techniques, reactive oxygen species, antioxidant methods, anti-inflammatory response, LC-MS

## Abstract

Mediterranean plants, such as fig and olive leaves, are well-known to exert beneficial effects in humans because of the presence of a wide range of bioactive compounds. However, scarce information regarding the impact of extraction methods, such as ultrasound and types of solvents, on their profile of antioxidant and anti-inflammatory compounds is provided. In addition, no information is available on the effects of extraction methods and solvents on the inhibition of pathogenic bacteria or promoting probiotic growth. In this scenario, this study was aimed to study the effects of ultrasound-assisted extraction (UAE) and solvent on the phenolic profile (Triple TOF-LC-MS/MS), antioxidant and anti-inflammatory compounds of olive and fig leaves. Results showed that UAE extracted more carotenoids compared to conventional extraction, while the conventional extraction impacted on higher flavonoids (olive leaves) and total phenolics (fig leaves). The antioxidant capacity of aqueous extract of fig leaves was three times higher than the extract obtained with ethanol for conventional extraction and four times higher for UAE. In general terms, hydroethanolic extracts presented the highest bacterial growth inhibition, and showed the highest anti-inflammatory activity. In conclusion, these side streams can be used as sources of bioactive compounds for further development of high-added-value products.

## 1. Introduction

From ancient times, Mediterranean plants and trees have attracted the interest of human being due to their beneficial properties such as antiviral, anti-inflammatory, prevention of cardiovascular diseases, and improvement of lipid metabolism to reduce obesity [1]. Most of these health-related effects have been attributed to their high content in bioactive compounds, such as polyphenols and carotenoids [2,3,4]. Among the different Mediterranean fruits, olive and fig, especially their leaves, have attracted the consumer’s attention because of their potential use as a source of traditional medicines, food additives, and preservatives. Additionally, some research studies showed that these materials can be used in nutrition and pharmaceutical industries [5,6]. As has been reported, fig leaves contain considerable amounts of antioxidants, especially phenolic compounds [1,5]. Oleuropein, hydroxytyrosol, luteolin-7-O-glucoside, verbascoside and apigenin-7-O-glucoside are the most abundant compounds and all display biologic activities including antioxidant, antimicrobial and antiproliferative properties [7]. Their chemical composition makes them well known for their therapeutic and medicinal properties for a long time showing great benefits on metabolism [8].

**Figure 1:**
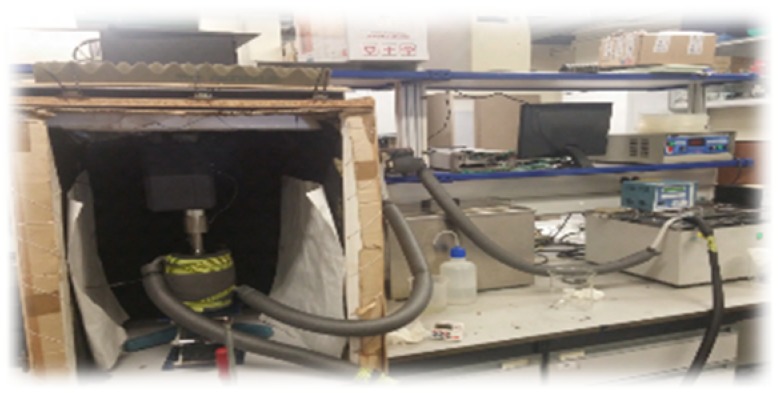
Experimental set-up for ultrasound assisted extraction.

The daily consumption of the antioxidant compounds from olive and fig leaves may reduce the risk of non-communicable diseases, such as cardiovascular diseases by inhibiting in vivo oxidation of low density lipoproteins [9,10]. Therefore, at this stage of development, improving/intensification of extraction processes coupling novel technologies, such as ultrasound, to conventional treatments is of paramount importance for consumers and food/pharmaceutical companies. Ultrasound assisted extraction (UAE) allows either avoiding or minimizing the use of organic solvents to extract high-added value compounds, along with other beneficial properties including the reduction of treatment time, intensification of heat and mass transfer transport, increasing the extraction yields, better preserving high extract quality, and reducing the energy consumption [11,12]. Thereby, ultrasound assisted extraction (UAE) may involve energy, solvent and time savings, which have positive implications not only on process productivity and cost reduction but also on environmental impact (Figure 1).

For instance, the studies evaluating the use of UAE at mild temperatures (<40 °C) from Mediterranean plant matrices is limited. In fact, there is only one previous study addressing the impact of UAE of polyphenols from olives leaves below room temperature [13]. The effect of UAE in liquid media is mainly attributed to cavitation phenomena, thus promoting stirring, and also to temperature increase associated to gas bubble implosion. However, although high temperatures could promote polyphenols extraction by increasing diffusion and solubility it could promote the degradation of thermolabile compounds [13,14]. Moreover, the phenomena linked to UAE are largely dependent on the extraction solvent used, which may also modify the phenolic profile of the extract as well as its bioactivity. In this regard, this works aims to assess the effect of conventional and ultrasound extraction at mild temperatures on the profile of antioxidant compounds of the different extracts obtained from dried fig and olive leaves using aqueous or hydroethanolic mixtures. Moreover, the impact of these extracts on the antioxidant capacity and anti-inflammatory response as well as their effect on bacterial growth, either pathogenic or potential beneficial bacteria will be evaluated.

## 2. Results and Discussion

### 2.1. Contents of Total Phenolics, Flavonoids, and Carotenoids

**Figure 2:**
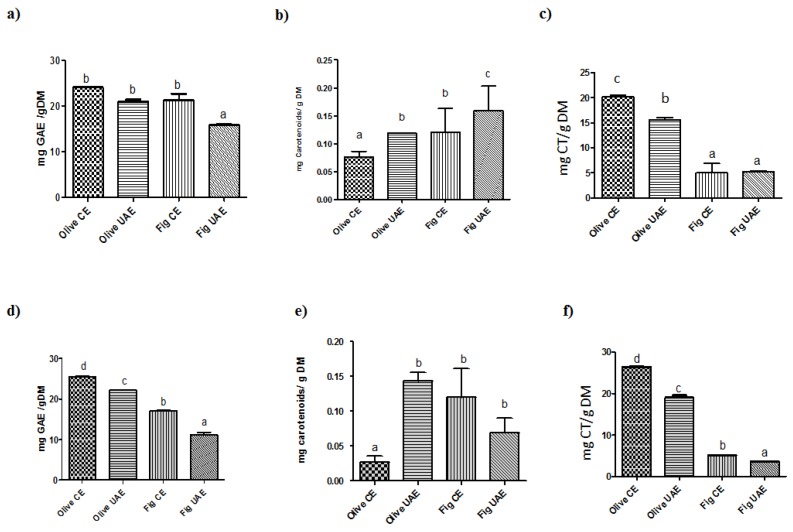
Content of total phenolics, flavonoids and carotenoids in fig and olive leaves extracts after conventional (CE) or ultrasound-assisted (UAE) extraction using either aqueous (**a**–**c**) or hydroethanolic (**d**–**f**) solvents. Total phenolic content (mg gallic acid equivalents (GAE)/g dry matter (DM)) (**a**,**d**), total flavonoid content (mg catechin (CT)/g DM) (**b**,**e**) and the carotenoids content (mg carotenoids/g DM sample) (**c**,**f**). Bars represent mean and standard error. Different letters comparing treatments represent statistically different mean values (*p* < 0.05).

The analysis of total phenolic content of different plant extracts obtained suggested that conventional extraction was more effective compared to an ultrasound assisted extraction regardless of solvent type (Figure 2).

In particular, results showed a higher total phenolic content for conventional extraction than for UAE. Our data displayed that the total phenolic content varied widely and ranged from 21.0 to 25.4 mg GAE/g DM for olive leaves and 11.1 to 21.2 mg GAE/g DM for fig leaves, taking into consideration two extraction techniques and two types of solvent. The highest total phenolic content was obtained by the conventional method in both plant extracts. Regardless of the solvent type, olive leaves showed higher total phenolic content when extracted with hydroethanolic solutions for both CE (25.4 mg GAE/g DM) and UAE (22.2 mg GAE/g DM) procedures, which is lower than the results available in the literature for UAE [15,16,17]. The total phenolic content obtained with water using the CE was 24.1 mg GAE/g DM, a concentration that was in line with values reported in the literature [18]. For fig leaves, higher total phenolic contents were obtained by aqueous extraction, both by CE and UAE (21.2 mg GAE/g DM versus 17.1 mg GAE/g DM in water-CE and hydroethanolic-CE method, respectively). For UAE, the total phenolic content was lower: 15.8 mg GAE/g DM in water and 11.128 mg GAE/g DM in hydroethanolic solution. Lower contents were recently described by Mopuri, Ganjayi, Meriga, Koorbanally, and Islam [19].

The total flavonoids content was measured in both hydroethanolic and aqueous extracts of olive and fig leaves with ultrasound assisted and conventional extractions (Figure 2). Plant extracts obtained with the CE presented a higher (*p* < 0.05) total flavonoids content than extracts obtained with UAE, except for fig leaves where with UAE water extracts showed slightly higher values (5.2 mg CT/g DM) than in ethanol (3.7 mg CT/g DM). Hydroethanolic extracts obtained with the CE method showed a concentration of 5.1 mg CT/g DM in ethanol and 5.0 mg CT/g DM in water. Total carotenoids content was influenced by the extraction method, mainly by the UAE extraction, especially for fig leaves which aqueous extracts had shown the significant highest level of 0.16 mg carotenoids/g (Figure 2). Regarding CE, no significant difference between two solvents was found (*p* > 0.05) in fig leaves. On the other hand, the extracts obtained with UAE presented higher total carotenoid content for olive leaves (0.12 mg carotenoid/g DM in water and 0.14 mg carotenoids/g DM in ethanol).

Although the benefits of ultrasound assisted extraction are well established in the previous literature, the results reported here confirm that its performance is strongly dependent on the process parameters used. Furthermore, ultrasound application is not efficient for every plant equally, as well as for extraction of certain compounds. The used process parameters were chosen from the literature research of optimal conditions for the highest values of extracted compounds [13,15]. Ultrasound exerts different phenomena when applied at high power in a liquid medium. The main effect of UAE is linked to the cavitation of air bubbles which involves large local release of mechanical and thermal energy into the medium, creating high local turbulence and temperature rise. In addition, alternating compression and expansions produced by ultrasound wave when propagating through the liquid bulk also increase turbulence [13,15]. Thereby, ultrasound application mainly reduces external resistance to mass transport improving the contact between the solvent and the sold matrix. In addition, it has also been reported the ultrasound ability to release components strongly attached to the solid matrix as well as to speed-up the molecular internal diffusion.

### 2.2. Individual Phenolic Composition, Antioxidant Capacity and Anti-Inflammatory Effects

**Figure 3:**
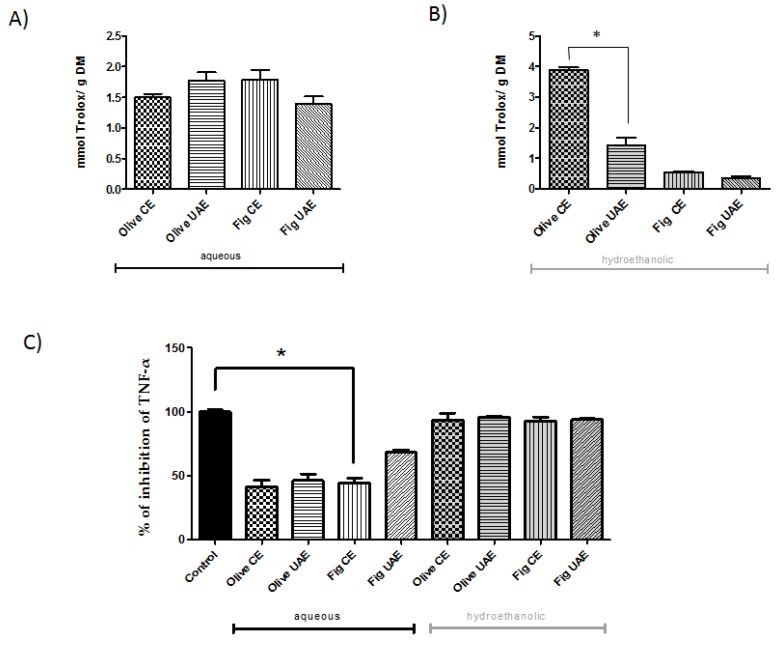
Antioxidant capacity and anti-inflammatory effects of the fig and olive leaves extracts. Antioxidant capacity (TEAC; Trolox Equivalent Antioxidant Capacity (mmol Trolox/g dry matter)) in (**A**) aqueous and (**B**) hydroethanolic (50:50, v/v, ethanol: water) extracts. (**C**) The effect of plant extracts on the TNF-α inhibition determined in olive and fig leaves after conventional (CE) or ultrasound-assisted (UAE) extraction. Differences (*p* < 0.05) between treatments are denoted by *.

Olive leaves were rich in total phenolic content and demonstrated a good antioxidant capacity. This is mainly caused by phenolic compounds such as oleuropein, hydroxytyrosol and verbascoside (and derivatives), detected in the aqueous extract (Table 1) and to a much lesser extent by, for instance, tocopherols. The antioxidant capacity results are reported in Figure 3A. 

In particular, extracts obtained with the conventional method presented a higher antioxidant activity compared to the extracts obtained with UAE. Among the plants analyzed, olive leaves extracted with ethanol/water mixture in the CE way showed the largest antioxidant activity (7.8 mmol TE) which was indicated by their high level of phenolic compounds, so the processing of olives changes the profile of phenolic compounds and therefore, both the organoleptic properties and the antioxidant capacity of the product.

The aqueous extract of fig leaves showed an antioxidant capacity three times higher than the extract obtained with ethanol for conventional extraction and four times higher for UAE. This might be attributed to the different polarity of water, thus modifying the solubility of the different target compounds [3,20]. Fig leaves presented the lowest antioxidant activity compared to the olive leaves (Figure 2). The levels of phytochemical phenolics and flavonoids compounds found in figs are strongly influenced by various factors such as the color, the part of fruit, the maturity and the drying process. Gallic acid, chlorogenic acid, quercetin-3-rutinoside and (−)-epicatechin are the most predominant phenolic acids and flavonoids in dried and fresh fig varieties [21] (Table 2).

The anti-inflammatory activity of fig/olive leaves extracts are shown in Figure 3C and it is possible to observe that the hydroethanolic extracts presented higher inhibition of TNF-α compared to the aqueous extracts. The high content of polyphenols in these extracts can easily explain this observation as these compounds seem to modulate the secretion of pro-inflammatory markers [22]. Peyrol, Riva, and Amiot [23] reported that the conversion of oleuropein into hydroxytyrosol (HT) has been associated to health benefits like the improvement of lipid and glycaemia profile and also the reduction of inflammatory processes and oxidative stress. HT is the major anti-inflammatory compound in aqueous olive extracts in inflammatory response induced by LPS in macrophages (mediated by inhibition of NO production, diminished secretion of cytokines and chemokines [24]. The content of HT in aqueous CE and UAE olive extracts (766 ± 26 and 646 ± 13 mg/kg, respectively) could explain the anti-inflammatory effect observed in our cellular model of inflammation. 

The two main compounds present in aqueous leaves fig extracts, apigenin and quercetin are flavonoids (a flavone and flavonol, respectively), strongly related to anti-inflammatory activity. Apigenin, a flavonoid more abundant in the aqueous extract of fig leaves (Table 3) is also found in parsley and celery and it has been described that it inhibits the LPS-induced pro-inflammatory cytokines expression by inactivating NF-κB. Moreover, the intake of apigenin also showed immunomodulation effects triggered by TNF-α in a mouse model of rheumatoid arthritis [25]. Quercetin, a ubiquitous plant secondary metabolite, is found abundant in onions, broccoli, apples, grapes, wine, tea, and leafy green vegetables, is well known as a potent antioxidant and anti-inflammatory agent. In aqueous extracts, this flavonol was very abundant (Table 3) and could be exerting part of the anti-inflammatory effect observed in the cell line HT-29 clone #16.

### 2.3. Effect of Extracts on Bacterial Growth

Different effects on bacterial growth were observed for the extracts, which seem to depend on the plant, solvent, methods and bacterial strain used (Table 3 and Table 4). 

Regardless of the extraction method, only the hydroethanolic extracts had antibacterial effect against *Salmonella enterica*. The specific growth rate was reduced between 35%–29% in presence of CE and UAE hydroethanolic extract, respectively (Table 3). Additionally, the DOmax obtained from the antimicrobial effect of leaves olive extract against *Salmonella enterica* can be explained by the presence of numerous bioactive compounds such as phenolic compounds (oleuropein, verbascoside, quercetin-3-rutinoside, luteolin 7-glucoside). The main phenolic compounds detected in olive leaves was oleoside, which could exert an important antibacterial effect as demonstrated by Medina, Romero-Gil, Garrido-Fernández, and Arroyo-López [26]. This study reported the survival of pathogens (*Escherichia coli*, *Staphylococcus aureus*, *Listeria monocytogenes*, and *Salmonella enterica*) in olive brines, and described that the most influential phenols on microbial survival were EDA (dialdehydic form of decarboxymethyl elenolic acid), HyEDA (EDA linked to hydroxytyrosol), hydroxytyrosol 4-glucoside, tyrosol, and oleoside 11-methyl ester. In addition, a review of sources and biological activities of rhoifolin summarized the effect antimicrobial of this flavonoid compound against *E. coli* [27]. This is another of the major components of olive leaves (6932 ± 574 and 61,230 ± 429 mg/kg, for CE and UAE extraction method, respectively) that may be exerting inhibition against *S. enterica*. 

Regarding the impact of plant extract on the growth of potential beneficial bacteria, it was observed an improvement of the *Lactobacillus casei* growth in aqueous extract of fig leaves, whereas no impact was found for *Bifidobacterium lactis* growth (Table 4). Many examples have been described of the antibacterial activity of flavonoids (such as apigenin and quercetin), which were extracted in relative high concentrations of fig leaves [28,29,30,31]. But little has been studied of a prebiotic effect of these compounds [32]. Some studies show that polyphenols can stimulate commensal and beneficial microbiota growth, while pathogenic strains can be inhibited [33]. In addition, Duda-Chodak [34] demonstrated that flavonoid aglycones, but not their glycosides, may inhibit growth of some intestinal bacteria. In this study also was observed slight stimulation of the growth of *Lactobacillus* spp. by quercetin-3-rutinoside. The extract of fig leaves contains quercetin-3-rutinoside at a high concentration (5008 ± 504 and 3539 ± 114 mg/kg, for CE and UAE methodology, respectively) that can explain the enhanced growth of *L. casei*. It has been suggested that several hydrolyzed and/or derived products from the catabolism of polyphenols by intestinal bacteria could exert both their physiological functions in the digestive tract, as well as their prebiotic properties and their modification of the gut microbiota [35].

## 3. Materials and Methods 

### 3.1. Plant Materials 

Olive (*Olea europaea*) and fig (*Ficus carica*) leaves were collected from MonteVedat-Torrent (Valencia, Spain) in September 2017, dried in a tunnel microwave dryer (Shandong Adasen Trade Co, JN-100, Beijing, China) overnight for 12 h (1200 W, 70 °C), then milled and stored at room temperature until being analyzed. Drying conditions were selected according to previous works due to its effect on antioxidant bioactive properties [36,37]. The authenticity of the plant materials was confirmed by the evaluation of the morphological structure of the leaves made by experts in the Department of Plant Biology of the University of Valencia, Spain.

### 3.2. Chemical Reagents

HPLC-grade solvents (acetonitrile, methanol and formic acid), Folin–Ciocalteu reagent, gallic acid, (+)-catechin, ABTS radical 2,2′-azinobis-(3-ethylbenzothiazoline-6-sulfonic acid), Trolox (6-hydroxy-2,5,7,8-tetramethylchroman-2-carboxylic acid, 97% purity) were purchased from Sigma–Aldrich (St. Louis, MO, USA). De Man, Rogosa, and Sharpe (MRS) agar and broth were acquired in Sigma-Aldrich (Darmstadt, Germany). All other chemical reagents were of analytical grade. 

### 3.3. Solvents and Extraction Methodology

Extraction experiments were carried out using two approaches: (1) distilled water (100%, v/v) and (2) hydro-alcoholic solution (50%, v/v). A ratio of 2% (w/v) and a total volume of 400 mL of solvent were used for each experiment and by use of two methods: (1) UAE and (2) conventional extraction (CE). It is noteworthy that the plant material:solvent ratio used in the current research is a preliminary experiment. Thus, any exploitation of the plant materials should be performed under more competitive conditions, e.g., at higher temperature or under UAE using flow-reactors.

UAE experiments were conducted using an ultrasonic probe system (UP400S, Dr. Hielscher, Teltow, Germany) at a constant temperature of 40 °C. Extraction time was fixed at 10 min according to the conditions previously described [7]. CE experiments were carried out in the same experimental conditions but replacing the ultrasonic tip by a mechanical stirrer (F 20520162, VELP Scientifica, Usmate Velate, Italy) at 1200 rpm. The experiments were performed according to the set-up previously established [38].

### 3.4. Total Phenolic, Flavonoid and Carotenoids Contents

The total phenolic content was estimated spectrophotometrically by the Folin–Ciocalteu reaction with some modifications [39]. Total phenolic content was expressed as milligrams of gallic acid equivalent per gram of dry plant extract (mg GAE/g DM). The identification and quantification of the major phenolic compounds present in the olive and fig leaves was carried out on TripleTOF™ 5600 (AB SCIEX) LC-MS/MS system equipped with Agilent 1260 Infinity (Agilent, Waldbronn, Germany). The MS acquisition was performed in the negative mode in the range between 80 and 1200 *m/z*, following the experimental procedure and methodology described in our previous work [38]. The flavonoid content was determined according to Sakanaka, Tachibana, and Okada [40]. (+)-Catechin was used for the analytical curve. The results were expressed as mg of catechin equivalent (CT) per gram of dry plant extract (mg CT/g DM). The extraction and quantification of total carotenoids was carried out in accordance to Lee and Castle [41]. All the analyses were performed in triplicate. 

### 3.5. Antioxidant Capacity and Anti-Inflammatory Effects 

The antioxidant activity was determined using the ABTS method [42]. The results, obtained from triplicate analyses, were expressed as mmol Trolox equivalent per gram of dry plant extract—mmol Trolox/g. The in vitro anti-inflammatory properties of extracts were assessed using a cell-reporter plasmid pNiFty2-SEAP (Invivogen) in HT-29 cells (human colon adenocarcinoma). In brief, HT-29 reporter-cells were seeded at 70.000 cells/well in 96-well plates and grown 24 h before the experiment. To analyze the NF-κB activation, cells were stimulated with/without a pro-inflammatory signal with TNF-α (20%) in the presence or absence of extracts (10 µL from each extract). After 24 h of stimulation, SEAP (secreted alkaline phosphatase) activity was quantified using *p*-nitrophenyl phosphate, as phosphatase substrate, in the supernatant according to the manufacturer’s instructions (Thermo Scientific, Ref.: 37620). The yellow-colored reaction products were detected using a microplate reader (Multiskan Ascent, Thermo-Fischer Scientific, Waltham, Massachusetts, USA) at 414 nm and the results were expressed as % of inhibition of TNF-α.

### 3.6. Effect of Extracts on Bacterial Growth 

The effect of leaves extracts on the bacterial growth was checked by the use of specific strains selected in base of their potential beneficial properties as probiotics and other, based on their potential foodborne and pathogenic properties. The strains used probiotics were *Lactobacillus casei* BL23 and *Bifidobacterium lactis* NCC2818, whereas the potential foodborne bacteria were *Listeria innocua* CECT 910, *Salmonella enterica* CECT 4138 and *Staphylococcus aureus* CECT 86. Probiotic strains were grown in MRS and MRS + 0.05% L-cysteine respectively in anaerobic and static conditions to 37 °C during 20 h while the potential pathogens were growth in brain heart infusion (BHI) medium in aerobic conditions to 37 °C overnight. 

Bacterial growth was monitored in presence or absence of 20 μL of the different extracts. Overnight cultures for each strain were collected by centrifugation inoculated to a final optical density at 595 nm of 0.05 in a 200 µL of broth medium in a 96 well microtiter plates and incubated at 37 °C. Changes in optical density at 595 nm were registered in a POLARStar reader (BMG Labtech, Ortenberg, Germany) and strains growth data were modelled by using the Gompertz equation [43] in order to mathematically describe the microbial growth and compute the specific growth rate in the exponential phase and optical density in the stationary one.

### 3.7. Statistical Analyses

Results were expressed as means followed by the standard deviation (*n* = 3). Significant differences between the results were calculated by one-way analysis of variances (ANOVA). Tukey’s test was applied to compare the mean values [44]. All statistical analyses were performed using the software Statgraphics^®^ Centurion XV (Statpoint Technologies, Inc., Virgin Islands, VI, USA).

## 4. Conclusions

Fig and olive leaves extract properties were dependent on extraction methodology (conventional or ultrasonically-assisted) and the solvent (aqueous or hydroethanolic). The combination of those factors affected the total phenolic, flavonoid and carotenoids content as well as the antioxidant and anti-inflammatory properties as well as affected the growth of different bacterial strains. Moreover, it seems that the use of extracts obtained from fig and olive leaves could have the potential to kill two birds with one stone and addressing both the stimulation of commensal and beneficial microbiota growth such as Lactobacillus, while inhibiting growth of pathogenic strains. However, it would be necessary to study the appropriate combination of polyphenols, as it seems the activity on microbial growth differs according to the type of polyphenol. Further research is of paramount importance in order to address the scaling-up of the ultrasonic assisted extraction for industrial purposes. Main aspects to be analyzed are related to the configuration of the ultrasonic emitter and the extraction vessel, the increase of the ratio dried material-solvent and the design of the cooling system to keep mild temperatures during the ultrasonic assisted extraction.

## Figures and Tables

**Table 1:** Triple TOF-LC-MS-MS analysis of the polyphenols (mg/kg) in aqueous olive leaves extracts obtained with conventional (CE) and ultrasound-assisted (UAE) procedures.

Compound Name	Formula	Expected *m/z*	CE	UAE
Oleoside 11-methylester	C_17_H_24_O_11_	403.1246	18537 ± 151	18128 ± 290
Rhoifolin	C_27_H_30_O_14_	577.1563	6932 ± 574	6123 ± 429
Demethyloleuropein	C_24_H_30_O_13_	525.1614	5676 ± 317	4692 ± 143
Querc-3-O-gal-7-O-rhamnoside	C_27_H_30_O_16_	609.1461	3555 ± 319	2651 ± 137
Phloretin xylosyl-galactoside	C_26_H_32_O_14_	567.1719	4164 ± 222	3369 ± 124
3-Hydroxyphloretin 2-O-xylosyl-gluc	C_26_H_32_O_15_	583.1668	ND	956 ± 57
Kaempferol 3-rutinoside	C_27_H_30_O_15_	593.1512	ND	2682 ± 213
Kaempferol 3-O-sophoroside	C_27_H_30_O_16_	609.1461	3555 ± 319	2651 ± 137
Verbascoside	C_29_H_36_O_15_	623.1981	2692 ± 21	2250 ± 122
Apigenin 6-C-glucoside	C_21_H_20_O_10_	431.0984	1956 ± 61	1593 ± 100
1-Sinapoyl-2-feruloylgentiobiose	C_33_H_40_O_18_	723.2142	1622 ± 124	1601 ± 153
Isorhamnetin 7-O-rhamnoside	C_22_H_22_O_11_	461.1089	1457 ± 125	1083 ± 101
Hydroxytyrosol	C_8_H_10_O_3_	153.0557	766 ± 26	646 ± 13
Diosmin	C_28_H_32_O_15_	607.1668	649 ± 21	548 ± 36
Kaempferol 3-O-rhamnosyl-rhamnosyl-gluc	C_33_H_40_O_19_	739.2091	639 ± 32	616 ± 19
Hydroxytyrosol 1-O-glucoside	C_14_H_20_O_9_	331.1035	667 ± 81	671 ± 23
Protocatechuic acid 4-O-glucoside	C_13_H_16_O_9_	315.0722	595 ± 17	488 ± 17
Sinapoyl glucose	C_17_H_22_O_10_	385.114	488 ± 24	ND
Matairesinol	C_20_H_22_O_6_	357.1344	455 ± 27	506 ± 18
Kaempferol	C_15_H_10_O_6_	285.0405	474 ± 65	216 ± 50
*p-*HPEA-EA	C_19_H_22_O_7_	361.1293	350 ± 61	ND
3,4-DHPEA-EA	C_19_H_22_O_8_	377.1242	ND	929 ± 139
3-Methylcatechol	C_7_H_8_O_2_	123.0452	339 ± 58	ND
Quercetin 3-O-glucoside	C_21_H_20_O_12_	463.0882	294 ± 8	171 ± 28
Oleoside dimethylester	C_18_H_26_O_11_	417.1402	285 ± 9	201 ± 22
4-Hydroxybenzoic acid 4-O-gluc	C_13_H_16_O_8_	299.0772	185 ± 5	156 ± 11
*p*-Coumaric acid	C_9_H_8_O_3_	163.0401	170 ± 1	126 ± 23
Dihydroquercetin 3-O-glucoside	C_21_H_22_O_12_	465.1039	163 ± 16	117 ± 12
3-Sinapoylquinic acid	C_18_H_22_O_10_	397.114	111 ± 13	ND
Quercetin	C_15_H_10_O_7_	301.0354	46 ± 5	ND
Rosmadial	C_20_H_24_O_5_	343.1551	ND	79 ± 45

ND: Not detected. Querc-3-O-gal-7-O-rhamnoside: Quercetin 3-O-galactoside 7-O-rhamnoside. Gluc: glucoside.

**Table 2:** Triple TOF-LC-MS-MS analysis of the polyphenols (mg/kg) in aqueous fig leaves extracts obtained with conventional (CE) and ultrasound-assisted (UAE) extraction procedures.

Compound Name	Formula	Expected *m/z*	CE	UAE
Apigenin 6-C-glucoside 8-C-arabinoside	C_26_H_28_O_14_	563.1406	16475 ± 2471	11765.26 ± 1038
Apigenin 6-C-glucoside	C_21_H_20_O_10_	431.0984	886 ± 30	509 ± 112
Quercetin 3- rutinoside	C_27_H_30_O_16_	609.1461	5008 ± 504	3539 ± 114
Rhoifolin	C_27_H_30_O_14_	577.1563	860 ± 83	578 ± 29
3-Feruloylquinic acid	C_17_H_20_O_9_	367.1035	2115 ± 126	417 ± 63
4-Hydroxycoumarin	C_9_H_6_O_3_	161.0244	1280 ± 180	719 ± 157
Ferulic acid	C_10_H_10_O_4_	193.0506	515 ± 97	292 ± 47
Kaempferol 3-O-xylosyl-glucoside	C_26_H_28_O_15_	579.1355	864 ± 71	602 ± 86
Kaempferol 3-O-xylosyl-rutinoside	C_32_H_38_O_19_	725.1935	ND	238 ± 27
3-Sinapoylquinic acid	C_18_H_22_O_10_	397.114	1294 ± 73	395 ± 73
Sinapoyl glucose	C_17_H_22_O_10_	385.114	751 ± 162	485 ± 99
Kaempferol 3-O-rhamnoside	C_21_H_20_O_10_	431.0984	886 ± 30	509 ± 112
Kaempferol 3-O-rutinoside	C_27_H_30_O_15_	593.1512	570 ± 16	368 ± 31
Isorhamnetin 7-O-rhamnoside	C_22_H_22_O_11_	461.1089	511 ± 26	285 ± 37
p-Coumaroyl malic acid	C_13_H_12_O_7_	279.051	184 ± 25	ND
Resveratrol	C_14_H_12_O_3_	227.0714	177 ± 37	98 ± 37
Didymin	C_28_H_34_O_14_	593.1876	145 ± 18	ND
Chrysoeriol	C_16_H_12_O_6_	299.0561	128 ± 46	ND
Oleoside 11-methylester	C_17_H_24_O_11_	403.1246	128 ± 15	70 ± 24
4-Hydroxybenzoic acid 4-O-glucoside	C_13_H_16_O_8_	299.0772	104 ± 22	ND
Rosmadial	C_20_H_24_O_5_	343.1551	60 ± 26	101 ± 80
Protocatechuic acid 4-O-glucoside	C_13_H_16_O_9_	315.0722	187 ± 5	120 ± 14
Cyanidin 3-O-(6-succinyl-glucoside)	C_25_H_25_O_14_	548.1172	86 ± 13	39 ± 7
Dihydrocaffeic acid	C_9_H_10_O_4_	181.0506	29 ± 10	13 ± 5
Quercetin 3-O-glucosyl-rhamnosyl-glucoside	C_33_H_40_O_21_	771.1989	155 ± 138	154 ± 22

Note: ND: Not detected.

**Table 3:** Effect of olive and fig leaves extracts in the growth rate and maximal optical density of bacteria strain.

Condition	Composition	Method *	Specific Growth Rate (h^−1^)	‡MOD
*Salmonella enterica*				
Bacteria	water	-	0.432 ± 0.006 ^a^	1.407 ± 0.033 ^c^
Bacteria	EtOH	-	0.484 ± 0.047 ^bcd^	1.418 ± 0.037 ^c^
Olive leaves	water	CE	0.334 ± 0.060 ^a^	1.406 ± 0.064 ^c^
		UAE	0.398 ± 0.017 ^ab^	1.362 ± 0.006 ^bc^
	EtOH	CE	0.322 ± 0.039 ^a^	1.236 ± 0.002 ^ab^
		UAE	0.345 ± 0.005 ^ab^	1.187 ± 0.074 ^a^
Fig leaves	water	CE	0.515 ± 0.043 ^cd^	1.471 ± 0.008 ^c^
		UAE	0.559 ± 0.051 ^d^	1.457 ± 0.011 ^c^
	EtOH	CE	0.571 ± 0.014 ^d^	1.435 ± 0.024 ^d^
		UAE	0.550 ± 0.15 ^d^	1.369 ± 0.006 ^bc^
*Listeria innocua*				
Bacteria	water	-	0.252 ± 0.22 ^a^	1.269 ± 0.017 ^abc^
Bacteria	EtOH	-	0.248 ± 0.042 ^a^	1.385 ± 0.003 ^bcd^
Olive leaves	water	CE	0.316 ± 0.034 ^a^	1.262 ± 0.037 ^abc^
		UAE	0.274 ± 0.079 ^a^	1.208 ± 0.004 ^a^
	EtOH	CE	0.358 ± 0.058 ^a^	1.189 ± 0.013 ^bcd^
		UAE	0.348 ± 0.063 ^a^	1.254 ± 0.090 ^d^
Fig leaves	water	CE	0.184 ± 0.009 ^a^	1.383 ± 0.008 ^a^
		UAE	0.233 ± 0.034 ^a^	1.423 ± 0.058 ^ab^
	EtOH	CE	0.235 ± 0.040 ^a^	1.407 ± 0.033 ^cd^
		UAE	0.300 ± 0.056 ^a^	1.391 ± 0.013 ^bcd^
*Staphylococcus aureus*				
Bacteria	water	-	0.556 ± 0.002 ^a^	1.851 ± 0.064 ^a^
Bacteria	EtOH	-	0.561 ± 0.075 ^a^	1.882 ± 0.001 ^a^
Olive leaves	water	CE	0.580 ± 0.024 ^a^	1.939 ± 0.033 ^a^
		UAE	0.636 ± 0.016 ^a^	1.899 ± 0.013 ^a^
	EtOH	CE	0.755 ± 0.144 ^a^	1.970 ± 0.028 ^a^
		UAE	0.657 ± 0.031 ^a^	1.989± 0.069 ^a^
Fig leaves	water	CE	0.638 ± 0.074 ^a^	1.901 ± 0.031 ^a^
		UAE	0.587 ± 0.003 ^a^	1.857 ± 0.028 ^a^
	EtOH	CE	0.601 ± 0.010 ^a^	1.851 ± 0.012 ^a^
		UAE	0.585 ± 0.006 ^a^	1870 ± 0.045 ^a^

Note: EtOH = Ethanol: * Method: Conventional extraction (CE) and ultrasound-assisted extraction (UAE). ‡MOD: Maximal optical density measured at 595 nm. Different letters in the same column represent statistically significant differences (*p* < 0.05).

**Table 4:** Effect of olive and fig leaves extracts in the growth rate and maximal optical density of probiotic bacteria strain.

Condition	Composition	Method	Specific Growth Rate (h^−1^)	‡MOD
*Lactobacillus casei*				
Bacteria	water	-	0.392 ± 0.001 ^a^	2.797 ± 0.027 ^a^
Bacteria	EtOH	-	0.383 ± 0.003 ^a^	2.837 ± 0.018 ^ab^
Olive leaves	water	CE	0.398 ± 0.016 ^a^	2.857 ± 0.058 ^ab^
		UAE	0.388 ± 0.005 ^a^	2.884 ± 0.033 ^ab^
	EtOH	CE	0.383 ± 0.008 ^a^	2.904 ± 0.071 ^abc^
		UAE	0.398 ± 0.007 ^a^	2.852 ± 0.083 ^ab^
Fig leaves	water	CE	0.437 ± 0.002^b^	3.077 ± 0.023 ^c^
		UAE	0.441 ± 0.003^b^	3.024 ± 0.041 ^bc^
	EtOH	CE	0.390 ± 0.009 ^a^	2.873 ± 0.062 ^ab^
		UAE	0.389 ± 0.007 ^a^	2.885 ± 0.006 ^abc^
*Bifidobacterium lactis*				
Bacteria	water	--	0.234 ± 0.021 ^a^	2.451 ± 0.052 ^a^
Bacteria	EtOH		0.236 ± 0.016 ^a^	2.463 ± 0.095 ^a^
Olive leaves	water	CE	0.255 ± 0.008 ^a^	2.634 ± 0.081 ^a^
		UAE	0.223 ± 0.005 ^a^	2.461 ± 0.013 ^a^
	EtOH	CE	0.233± 0.011 ^a^	2.492 ± 0.047 ^a^
		UAE	0.229 ± 0.017 ^a^	2.481 ± 0.008 ^a^
Fig leaves	water	CE	0.252 ± 0.003 ^a^	2.481 ± 0.013 ^a^
		UAE	0.263 ± 0.007 ^a^	2.540 ± 0.036 ^a^
	EtOH	CE	0.217 ± 0.006 ^a^	2.386 ± 0.050 ^a^
		UAE	0.234 ± 0.005 ^a^	2.476 ± 0.012 ^a^

Note: EtOH = Ethanol: * Method: Conventional extraction (CE) and ultrasound-assisted extraction (UAE). ‡MOD: Maximal optical density measured at 595 nm. Different letters in the same column represent statistically significant differences (*p* < 0.05).
